# Difficulty in continuing home care after informal caregiver was exposed to the COVID‐19: A case report

**DOI:** 10.1002/ccr3.5804

**Published:** 2022-07-18

**Authors:** Ryo Sakamoto, Makoto Yoshida, Divya Bhandari, Hirotomo Miyatake, Makoto Kosaka, Tetsuya Tanimoto, Masahiro Kami, Akihiko Ozaki

**Affiliations:** ^1^ Visina Home Care Tokyo Japan; ^2^ Teikyo University school of Medicine Tokyo Japan; ^3^ 536425 Medical Governance Research Institute Tokyo Japan; ^4^ Orange Home‐Care Clinic Fukui Japan; ^5^ Department of Breast Surgery Jyoban Hospital of Tokiwa Foundation Fukushima Japan

**Keywords:** health maintenance, homecare, infectious diseases, nursing, social care

## Abstract

Home care can present many challenges without management. During COVID‐19 pandemic, when an informal caregiver becomes infected and had to isolate themselves, finding another caregiver becomes extremely challenging. For terminally ill patients, who relies on other for even minor tasks, interruption of care could pose severe negative consequences.

## Abstract 2

In home care settings, when informal caregivers are exposed to COVID‐19, it may be difficult to find replacement caregivers and care could be interrupted.

## 1 INTRODUCTION

Although chronic and terminally ill patients have been usually cared for in nursing homes, people are increasingly choosing home care as an alternative type of medical care,^1^ Japan is no exception. Home care consists of public services that provide necessary medical and nursing care, such as home medical care by doctors, home nursing care by nurses and caregivers, along with family care. Home care in Japan is usually covered by public insurance such as medical insurance and long‐term care insurance.^2^ As a result, patients receiving home care are from diverse backgrounds, including those suffering from chronic or intractable diseases, terminal stages of life, and mental illness.

Due to visiting restrictions imposed during the coronavirus disease 2019 (COVID‐19) pandemic, patients admitted to a hospital are usually unable to see or communicate effectively with their family members.^3^, ^4^ Therefore, increasingly more patients are choosing home care to live their fulfilling life with their families at their end‐stage of life.^5^ Studies have reported the importance of end‐of‐life home care during COVID‐19.^6^ While it could seem an effective option, it is necessary to understand, acknowledge, and be prepared for possible challenges that may arise.

In this article, we have reported the challenges faced in continuing home care after informal caregiver was exposed to the COVID‐19, underscoring the need for effective planning and preparation for the anticipated and unanticipated future challenges before opting for home care.

## 2 CASE PRESENTATION

An elderly woman in her 80s with dementia was referred to our home nursing station in 2021. Originally, she used to run a restaurant with her husband but they closed it five years ago due to her health concerns. Her husband also passed away three years ago and she has been living alone since then. Normally, she used a daycare service six days a week from Monday to Saturday, where she received exercise, leisure activities, and cleanliness care including bathing assistance. In addition, she used to receive cleaning services and personal care from a home care service once a week, and her son also visited her almost every day. Meals were usually delivered to her home in a lunch box, and she was able to eat at the dining table. However, sometimes, due to short‐term memory loss, she used to miss her meal. To ensure she ate it properly, her daughter‐in‐law would call her and her son would visit her every morning. Although she was able to defecate on her own most of the time, there were a few times when she did not; so, the caregiver had to check her diapers and encourage her to change them.

In late June 2021, her son was found to be infected with SARS‐CoV‐2 and was admitted to a hospital. As she had been in close contact with him, the visiting physician immediately conducted a PCR test on her. Despite the test result being negative her usual home care and daycare services were completely suspended as she was in contact with an infected person. Furthermore, her daughter‐in‐law also did not feel comfortable visiting her. So, our institute was recommended by the public health center to a care manager who was seeking a home nursing station equipped to care for patients who had been in close contact with COVID‐19 patients. Our role was to manage home care until a short‐term accommodation could be found.

We made our first home nursing visit around noon, the day after her son's admission. We were informed in advance that she had dementia and she could refuse care services. However, we did not experience any rejection for care and appropriate precautions were taken to prevent COVID‐19 infection. When she learned her son had been admitted to the hospital, she broke down in tears. Upon calming her down, the nursing staff checked her vital signs, helped her take meals, and medication, and looked after her hygiene needs. After a few days, the care manager was able to find a private facility where she could stay for a short period. Besides helping her prepare for the stay, our staff took her to a cab where she was accompanied by her daughter‐in‐law.

## 3 DISCUSSION

This case study has highlighted a challenge that could arise in‐home care particularly when a caregiver is exposed to an infectious disease such as COVID‐19. While a caregiver was isolated due to COVID‐19 infection, a patient was left unattended by other family members and the care center as she was exposed to the infected person. The patient could have faced severe problems if we had not provided timely support. As mentioned in the case presentation, all her care services were suspended, and she was physically unable to take care of herself or even remember simple things like eating or excreting properly; if she had not received the timely care, it could have directly compromised her physical health and increased the risk of infection.

Long‐term care insurance in Japan is based on the premise of family care. However, when an informal caregiver becomes infected with SARS‐CoV‐2 or any other disease and is quarantined in a hospital or other institution, there would be no one to care for the patient as in this case. It is particularly difficult to find alternative caregivers when the patient is exposed to COVID‐19 infected person. Moreover, staffs of day‐care and home care facilities are not necessarily trained in medical countermeasures against infectious diseases, making it difficult for them to take care of those with close contact with a COVID‐19 patient.

In this case, the home nursing station staff visited the patient with infection control measures in place. For example, protective clothing was worn when entering the room, a separate nurse in charge was allocated for this case, and an office was set up exclusively for her. Thus, like in this case, in countries with home care systems, including Japan, it may be necessary to establish flexible rules for dealing with close contact with a COVID‐19 patient, such as allowing public services to intervene in home care with infection control.

Luckily, in this case, the patient was not in a critical condition. However, for terminally ill patients, who are usually in critical condition, disruption of care could pose a high risk to both physical and mental health. Home care became a necessity in Japan, particularly during COVID‐19 when there is a constant rise in cases, difficult hospitalization, and a shortage of health workers and beds.^6^ However, while emphasizing the importance of home care, it is equally important to expand home care measures, particularly during the COVID‐19 pandemic, as underlined by this case.

We must also study aspects of psychiatric to gain a comprehensive understanding of the impact COVID‐19 on healthcare delivery. COVID‐19 has led to isolation guidelines which has resulted in a lot of adverse effects in the mental health of the population by living in isolation and quarantine.^7^ COVID‐19 has also brought upon the worsening of various psychiatric conditions such as depression, anxiety, posttraumatic stress disorder, and obsessive compulsive disorder as noted in various studies.^8^, ^9^ Due to COVID‐19, the health systems and the healthcare workers experienced an unprecedented amount of stress leading to an increase in the number of suicide events in health care workers including physicians.^10^ Healthcare delivery across the globe had to be adjusted and refined as per the new requirements.^11^ Further study was needed to determine what psychiatric issues existed in home care in COVID‐19 pandemic.

## 4 CONCLUSION

This case presents the challenge faced in continuing home care once an informal caregiver was exposed to the COVID‐19. It is one of many challenges that can arise while providing home care to a terminally ill patient. While home care can be a viable option, it is necessary to make effective planning and be prepared for the anticipated and unanticipated future challenges. This holds true during COVID‐19 and beyond.

## AUTHOR CONTRIBUTIONS

All authors conceptualized and designed the study. Sakamoto R, Yoshida M, Bhandari D, Ozaki A, and Tanimoto T wrote the manuscript, and all authors contributed to making critical revisions for improving the intellectual content of the manuscript.

## CONFLICT OF INTEREST

AO reports personal fees from Medical Network Systems, MNES Inc. TT reports personal fees from Medical Network Systems, MNES Inc. and Bionics co. Itd. Other authors declare no competing interests.

## ETHICAL APPROVAL

This research meets the ethical guidelines and adheres to Japan's local legal requirements. An ethical review is not required for this type of article.

## CONSENT

Written informed consent was obtained from the patient to publish this report in accordance with the journal's patient consent policy.

## Data Availability

Data sharing is not applicable to this article as no datasets were generated or analyzed during current study.
